# Amino Acid Metabolites and Slow Weight Loss in the Early Postoperative Period after Sleeve Gastrectomy

**DOI:** 10.3390/jcm9082348

**Published:** 2020-07-23

**Authors:** Yeongkeun Kwon, Mi Jang, Youngsun Lee, Jane Ha, Sungsoo Park

**Affiliations:** 1 Division of Foregut Surgery, Korea University College of Medicine, Seoul 02841, Korea; kukwon@korea.ac.kr; 2 Center for Obesity and Metabolic Diseases, Korea University Anam Hospital, Seoul 02841, Korea; janehapti@korea.ac.kr; 3 Department of Biotechnology and Food Science, Norwegian University of Science and Technology, 7491 Trondheim, Norway; kia3111@gmail.com; 4 Advanced Analysis Center, Korea Institute of Science and Technology, Seoul 02792, Korea; youngsunlee@snu.ac.kr; 5 Department of Medicine, Korea University College of Medicine, Seoul 02841, Korea

**Keywords:** amino acid, metabolomics, sleeve gastrectomy, bariatric surgery, weight loss

## Abstract

Background: Profiles of amino acid metabolites (AAMs) have been linked to obesity and energy homeostasis. We investigated whether baseline obesity-related AAMs were associated with weight status in the early postoperative period after sleeve gastrectomy. Methods: In this prospective, single-arm, longitudinal study, 27 bariatric patients underwent sleeve gastrectomy. Twenty obesity-related AAMs were comprehensively quantified prior to surgery, and slow weight loss was defined as the lowest 40% of the percentage excess weight loss (%EWL) at three and six months postoperatively. Linear regression models were used to assess the association between baseline obesity-related AAMs and %EWL, and receiver operating characteristic curves were assessed. Results: Isoleucine and metabolites from the serotonin pathway were significantly associated with the %EWL at three and six months after sleeve gastrectomy. Among the metabolites identified to be significant in the regression analyses, serotonin (area under receiver operating characteristic curves (AUROC): 0.79, 95% confidence interval (CI): 0.59–0.97) and serotonin/5-hydroxytryptophan ratio (AUROC: 0.80, 95% CI: 0.58–1.00) showed superior performance in predicting slow weight loss six months after sleeve gastrectomy. Conclusions: Our findings underscore the importance of baseline AAM profiles, especially serotonin and serotonin/5-hydroxytryptophan ratio, in predicting slow weight loss in the early postoperative period after sleeve gastrectomy.

## 1. Introduction

Bariatric surgery is currently the most successful and durable treatment for the morbidly obese [1,2,3]; however, there is wide variability in the weight loss response to bariatric surgery [4,5,6] and over 20% of bariatric patients experience long-term postoperative weight regain [2,7,8]. Although suboptimal weight loss after bariatric surgery has been known to be associated with the recurrence of obesity-related comorbidities and a deterioration in the health-related quality of life [9,10,11], there currently exist few effective methods for predicting postoperative weight status. Recently, weight loss in the early postoperative period has been suggested to be able to predict long-term weight outcomes [12,13]. Indicators such as the percentage excess weight loss (%EWL) or weight loss velocity up to six months after bariatric surgery have been suggested to predict long-term weight response to bariatric surgery [12,13]. Given that postoperative behavioral or intensive lifestyle interventions improve weight loss after bariatric surgery [14,15], early identification of slow weight loss responders is an important focus area for individualized postoperative care.

Interestingly, circulating concentrations of branched-chain amino acids (BCAAs), aromatic amino acids (AAAs), and various tryptophan-derived metabolites (TDMs) have been highlighted as potential biomarkers for obesity-related medical conditions. BCAAs, AAAs, and various TDMs as signaling molecules participate in nutritional metabolism and energy homeostasis and the adipose tissue has been known to regulate these circulating metabolites [16,17,18,19]. Levels of circulating BCAAs and AAAs tend to be elevated in individuals with obesity and appear to be closely related to an individual’s metabolic health and future insulin resistance or type 2 diabetes [20,21]. Although several mechanisms (e.g., activation of mammalian target of rapamycin complex 1, mitochondrial dysfunction induced by amino acids dysmetabolism) have been proposed to explain the increased amino acid and insulin resistance and obesity, the exact causative associations have not been investigated [20]. Among various TDMs, peripheral serotonin has been well known as affecting organismal energy homeostasis [22,23], and inhibition of peripheral serotonin synthesis protects against diet-induced obesity [24]. Serotonin metabolism has been known to regulate glucose levels and in turn obesity through its effects on hepatocyte and adipocyte functions [25].

Sleeve gastrectomy is one of the most commonly performed bariatric surgeries and appears to be similar in achieve weight loss compared with the Roux-en-Y gastric bypass, which has been considered the gold standard procedure for morbidly obese patients [26]. To elucidate the role of AAMs as potential predictors for early postoperative weight status after sleeve gastrectomy, we performed a comprehensive metabolomic study targeting 20 obesity-related AAMs in bariatric patients and investigated whether pre-operative AAMs are associated with weight loss in the early postoperative period after sleeve gastrectomy.

## 2. Methods

### 2.1. Study Participants

In January 2019, a prospective, single-arm, longitudinal study to assess the effect of bariatric surgery on energy homeostasis began at a university hospital. The original study was designed to perform follow ups until 12 months postoperative, and this study details the interim results at six months postoperative (Institutional Review Board approval number: 2019AN0055). Following the general criteria for bariatric surgery in Korea, eligibility criteria included body mass index (BMI) ≥ 35 kg/m^2^, or BMI ≥ 30 kg/m^2^ and at least one or more obesity-related co-morbidities, and age ≥ 20. Patients were excluded if they had previous bariatric surgeries, other complex abdominal surgeries, or had poorly controlled medical or psychiatric disorders (details of eligibility criteria are presented in Supplementary Table S1). Because of the lack of literature on the association between AAMs and weight status after bariatric surgery, sample size calculation was not performed. We designed this study with a sample size of 30 participants out of whom three failed to report during follow-ups. Patients providing written informed consent entered a screening process for study eligibility and underwent physical and laboratory evaluations to confirm eligibility.

### 2.2. Pre-operative Education of Bariatric Patients

Following study enrollment, all patients received nutritional evaluations including weight-loss expectations, eating behaviors and patterns, physical activity habits, and psychosocial assessments. To achieve optimal weight loss after sleeve gastrectomy, a bariatric physician and registered dietitian provided dietary and lifestyle recommendations including dietary principles, such as macro- and micro-nutrient compositions, carbohydrate counting, and advice regarding regular aerobic exercise (if medically approved by the physician who provided their medical care). All patients began guideline-based micronutrient (vitamin and mineral) supplementation after enrollment in this study [27].

### 2.3. Surgical Procedures

Sleeve gastrectomies were performed laparoscopically by a single bariatric surgeon and involved a gastric volume reduction of 80% to 85% using a 30-French endoscope to perform stomach resections beginning 3 cm from the pylorus and terminating at the angle of His.

### 2.4. Postoperative Care Regarding Nutrition and Exercise

Postoperatively, patients received education via a protocol-driven staged-meal progression. If tolerable, a low-sugar clear liquid meal was initiated within 24 h after sleeve gastrectomy, and soft and regular diets were recommended to begin at three–four weeks and nine weeks, respectively. A protein intake of 50 g/day and up to 1.5 g/kg ideal body weight per day was recommended with oral supplementation of amino acids including BCAAs and AAAs: leucine 5.55 g/day, isoleucine 2.65 g/day, valine 2.7 g/day, phenylalanine 1.75 g/day, tyrosine 1.75 g/day, and tryptophan 1.35 g/day. Promax^®^ (Korea Medical Foods Co., Seoul, South Korea) was used for amino acid supplements.

In the first four weeks postoperatively, patients exercised by walking and gradually increased speed in their tolerated threshold. Patients were asked to walk 150 min per week. In 5–26 weeks postoperatively the patient’s total walking time increased to ≥ 200 min per week and ≥ 4 days per week. Additionally, patients were asked to perform three ≥ 20 min strength exercise sessions including shoulder and hip strengthening exercises ≥ 3 days per week. The intensity of the exercises was a perceived exertion rating between 12 to 14 on the Borg Scale [28]. All participants were followed-up every two weeks via telephone and by text message to confirm compliance with their nutritional and exercise recommendations.

### 2.5. Measurements of Serum AAMs

Pre-operative blood samples of patients were obtained within two weeks prior to surgery. Blood sampling was performed eight h after fasting when patients were not on a pre-operative calorie-restricted diet. Amino acid profiling was performed using liquid chromatography-mass spectrometry in the College of Life Sciences & Biotechnology, Korea University, Seoul, Korea. We selected 20 obesity-related AAMs based on the results of previous studies relating AAMs and obesity or energy homeostasis (Supplementary Figure S1): (1) BCAAs (leucine, isoleucine, valine); (2) AAAs (phenylalanine, tyrosine, tryptophan); (3) TDMs including kynurenine pathway metabolites (kynurenine, anthranilic acid, 3-hydroxykynurenine, 3-hydroxyanthranilic acid, kynurenic acid, xanthurenic acid); indole pathway metabolites (indoxyl sulfate, indole-3-acetic acid, indole-3-lactic acid, indole-3-propionic acid); serotonin pathway metabolites (5-hydroxytryptophan (5-HTrp), serotonin, 5-hydroxyindoleacetic acid (5-HIAA)); and tyrosine pathway metabolites (L-dihydroxyphenylalanine). We also calculated the ratios between adjacent metabolites (downstream metabolites/upstream metabolites) to compare the enzymatic activity among participants. A detailed protocol for serum metabolite measurements and enzymes corresponding to the metabolite ratios are presented in Supplementary Tables S2 and S3.

### 2.6. Outcome Measures

The primary outcomes were the associations between baseline obesity-related AAMs and changes in weight status three and six months post sleeve gastrectomy. Changes in weight status were calculated with %EWL by dividing the number of kilograms lost by the number of kilograms in the patient’s excess body weight.

### 2.7. Statistical Analysis

Summary data are presented as percentages for categorical variables and as means with standard deviations (SDs) for continuous variables. Patients’ characteristics at baseline and three or six months postoperatively were compared using the paired t-test or Wilcoxon signed rank test for continuous variables. Serum metabolite concentrations were log-transformed to improve the normality of their distributions based on the Shapiro-Wilk test results. First, we examined associations of %EWL with baseline obesity-related AAMs three and six months postoperatively using linear regression models adjusted for baseline BMIs. Sensitivity analyses were also performed to determine the consistency of the statistical significance according to the following baseline characteristics: age ≥ 45 years, female sex, BMI ≥ 35 kg/m2, hypertension, diabetes, and non-smoker status. Secondly, using AAMs identified as significant in the regression analyses, receiver operating characteristic (ROC) curves were generated to analyze individual AAM performances to predict slow weight loss three and six months postoperatively. Considering that about 40% of the bariatric patients at our hospital do not attain 50% EWL at 6 months postoperative, slow weight loss was defined as the lowest 40% of the %EWL at three and six months postoperative. Statistical analyses were performed using Stata12 (Stata Corp., College Station, TX, USA), and a two-sided *p*-value of < 0.05 was considered statistically significant.

## 3. Results

### 3.1. Patients’ Characteristics at Baseline

The mean age of the 27 study participants was 42.1 years (SD: 12.9), and 63% of the patients were women (Table 1). The mean BMI and mean waist circumference were 38.7 kg/m^2^ (SD: 5.2) and 120.5 cm (SD: 19.9), respectively. Among the study participants, 78% had metabolic syndromes and 15% were current smokers. Participants with hypertension, diabetes, and dyslipidemia were 52%, 74%, and 74%, respectively.

### 3.2. Changes in Patients’ Characteristics after Bariatric Surgery

All patients experienced a significant decrease in BMI and body weight (BMI at three months: 31.5 kg/m^2^ (SD: 5.1), *p* < 0.001; body weight at three months: 85.1 kg (SD: 15.3), *p* < 0.001; BMI at six months: 27.9 kg/m^2^ (SD: 4.6), *p* < 0.001; and body weight at six months: 73.9 kg (SD: 11.5), *p* < 0.001) (Table 2). Patients presented with a mean of 62.5% (SD: 35.1) and 92.3% (SD: 49.8) %EWL at three and six months after bariatric surgery, respectively. Body fat mass (BFM) and fat free mass (FFM) also showed significant postoperative decreases at three months (BFM: *p* < 0.001; FFM: *p* < 0.001) and six months (BFM: *p* = 0.002; FFM: *p* = 0.007). Fasting plasma glucose levels decreased to 14.5% of the baseline at three months (*p* = 0.028), and 17.0% of the baseline at six months (*p* = 0.034). Patients’ homeostatic model assessment of insulin resistance (HOMA-IR) improved at three months (2.8 (SD: 1.6), *p* = 0.033) and at six months (2.2 (SD: 0.6), *p* = 0.044) compared to the baseline. Mean systolic and diastolic blood pressures at six months were 126.1 mmHg and 83.1 mmHg, respectively, which represented a decrease of 11.6% and 14.9% from the baseline, respectively. Mean high-density lipoprotein (HDL) cholesterol and triglyceride levels at six months were 54.6 mg/dL and 115.6 mg/dL, respectively, which represented a 15.6% increase from the baseline (*p* = 0.041) and a decrease of 23.6% from the baseline (*p* = 0.016), respectively.

### 3.3. Baseline AAMs and Weight Loss after Sleeve Gastrectomy

Several baseline AAMs were associated with %EWL at three and six months after sleeve gastrectomy (Table 3). Baseline isoleucine was significantly associated with %EWL at three months (β (standard error (SE)): 0.67 (0.27); *p* = 0.026) and six months (β (SE): 1.02 (0.36); *p* = 0.016) postoperatively. Among metabolites from the serotonin pathway, 5-HIAA and 5-HIAA/serotonin ratio were significantly associated with %EWL at three months (serotonin: β (SE), −39.7 (13.5); *p* = 0.009; 5-HIAA: β (SE), 106.7 (32.5); *p* = 0.004; and 5-HIAA/serotonin ratio: β (SE), 54.7 (12.4); *p* < 0.001), and at six months (serotonin: β (SE), −50.8 (21.1); *p* = 0.032; 5-HIAA: β (SE), 125.2 (54.7)O; *p* = 0.040; and 5-HIAA/serotonin ratio: β (SE), 54.5 (21.6); *p* = 0.026) post bariatric surgery. The 5-HTrp/tryptophan ratio (β (SE): 64.6 (30.6); *p* = 0.049) and the serotonin/5-HTrp ratio (β (SE): −45.7 (14.1); *p* = 0.005) were significantly associated with %EWL at three months, but not at six months post bariatric surgery. The results of other AAMs are presented in Supplementary Table S4. Sensitivity analyses showed that the statistical significance did not change in the various patient groups (Supplementary Table S5).

### 3.4. Prediction for Slow Weight Loss after Sleeve Gastrectomy

**Figure 1:**
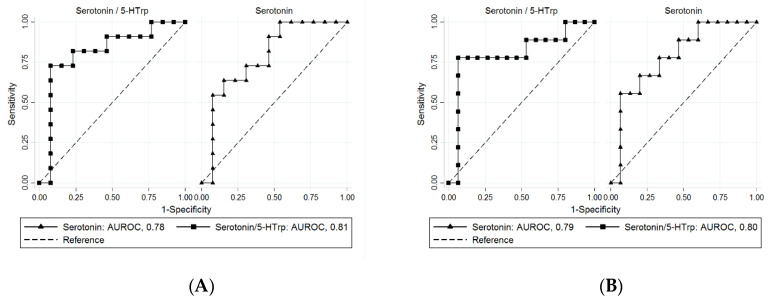
Performance of baseline serotonin and serotonin/5-HTrp ratio in predicting slow weight loss at: (**A**) three months and (**B**) six months after sleeve gastrectomy. AUROCs were calculated using amino acid metabolites proven to be significant (Table 3). Among the test metabolites, serotonin and serotonin/5-HTrp ratio showed superior prognostic performance with the best discriminatory ability for predicting slow weight loss three and six months after sleeve gastrectomy. AUROC values included: serotonin, 0.78 (95% CI: 0.58–0.97) at three months and 0.79 (95% CI: 0.59–0.97) at six months; serotonin/5-HTrp ratio, 0.81 (95% CI: 0.61–1.00) at three months and 0.80 (95% CI: 0.58–1.00) at six months. 5-HTrp: 5-hydroxytryptophan, AUROC: area under receiver operating characteristic curves, CI: confidence interval.

ROC curves were generated with isoleucine and metabolites from the serotonin pathway, which were significantly associated with %EWL after sleeve gastrectomy (Table 3). Serotonin and serotonin/5-HTrp ratio showed superior performance in predicting slow weight loss at three and six months postoperatively (Figure 1). The values of the AUROCs were, serotonin, 0.78 (95% CI: 0.58–0.97) at three months and 0.79 (95% CI: 0.59–0.97) at six months; serotonin/5-HTrp ratio, 0.81 (95% CI: 0.61–1.00) at three months and 0.80 (95% CI: 0.58–1.00) at six months.

## 4. Discussion

In this study, we showed that the profiles of obesity-related AAMs before sleeve gastrectomy were significantly associated with %EWL at three and six months postoperatively. Among the AAMs which proved to be significant, serotonin and the serotonin/5-HTrp ratio showed superior prognostic performance with the best discriminatory ability for slow weight loss at three and six months after sleeve gastrectomy. To our knowledge, these results are the first to suggest that pre-operative AAM profiles are useful biomarkers for predicting early postoperative weight status after sleeve gastrectomy.

Our findings, which highlight BCAAs, are noteworthy in the context of experimental and clinical data which suggest that BCAAs may be markers of insulin resistance in obesity [21,29,30]. Although changes in the levels of amino acids, including BCAAs and AAAs were observed in patients who underwent bariatric surgery [31,32,33], less knowledge is available regarding how pre-bariatric surgery amino acid profiles affect weight loss after bariatric surgery. Our data showed that pre-operative BCAA profiles, especially higher levels of serum isoleucine, were associated with more successful weight loss in the relatively early postoperative period (three and six months postoperatively) (Table 3). Given that isoleucine has been known to have a role in the improvement of visceral obesity and hyperinsulinemia and lipid metabolism in white adipose tissue [34,35], our findings support the opinion that hyper-isoleucinemia could be a pre-operative manifestation to predict optimal weight loss after sleeve gastrectomy.

Our results, which underscore serum metabolites from the serotonin pathway for predicting slow weight loss after sleeve gastrectomy, should also be viewed in the context of previous studies suggesting peripheral serotonin as a potential biological mediator in energy homeostasis [4,24]. Since serotonin cannot cross the blood-brain barrier, central and peripheral serotonin systems are functionally separated and serotonin is synthesized from the essential amino acid tryptophan by the sequential actions of tryptophan hydroxylase (TPH) and aromatic L-amino acid decarboxylase (AADC) (Supplementary Figure S1). Increased circulating serotonin levels are observed in mice with diet-induced obesity [22,36] and humans with obesity [37]. Additionally, gain-of-function polymorphisms in TPH, which promote hyperserotonemia, were associated with BMI and waist circumference in a genome-wide association study of nondiabetic individuals [38]. However, the absence of serotonin through a genetic or pharmacological block of peripheral TPH protects against the development of metabolic syndrome in mice on a high-fat diet [24,39].

Our results showed that lower levels of serotonin and higher levels of 5-HIAA before surgery were associated with higher %EWL at three and six months postoperatively (Table 3). In accordance with these results, lower level of serotonin/5-HTrp ratio (representing AADC activity) and higher level of 5-HIAA/serotonin ratio (representing monoamine oxidase A (MAO-A) activity), which promote hyposerotonemia, were associated with higher %EWL at three months postoperatively. Serotonin and serotonin/5-HTrp ratio in particular showed superior performance in predicting slow weight loss three and six months after sleeve gastrectomy (Figure 1). Circulating serotonin has been known to interact with multiple organs and stimulate insulin secretion and lipogenesis, thereby accelerating the energy storage process of the body [40]. Our findings on the association between hyposerotonemia and rapid postoperative weight loss is in line with previous studies that have demonstrated that peripheral serotonin can promote efficient energy storage. Our results are the first to suggest that serum metabolites from the serotonin pathway predict weight loss after sleeve gastrectomy and further studies are warranted to assess whether the serotonin pathway contributes to a variability in the weight loss response.

This study had several limitations. First, our results did not preclude that other serum metabolites may also predict weight status after sleeve gastrectomies. For example, analyzing downstream kynurenine pathway metabolites such as quinolinic acid and nicotinamide adenine dinucleotide, and BCAA metabolites such as alanine, glutamine, and glutamate would require further evaluation. Secondly, postoperative energy balance, mainly determined by caloric intake and expenditure, could also affect postoperative weight status. Although we monitored compliance and postoperative diet and exercise recommendations every two weeks, bias could occur due to indirect supervision via telephone or text messages. Thirdly, supplementation of amino acids may also modify the alteration in postoperative weight status [41,42]. However, we followed and monitored the equivalent intake of oral amino acid supplements during the study and various sensitivity analyses showed a consistency of statistical significance for the overall results. Fourthly, our study was conducted on a small number of Asian patients with relatively low BMIs and high rates of metabolic syndrome (the mean BMI was 38.7 kg/m^2^ and 78% of the patients had metabolic syndrome). Caution is advised in applying our results to patients of other ethnicities, patients with higher BMIs, or patients with lower rates of metabolic syndrome. Our results should be further validated in studies with more patients. Fifthly, as an early postoperative study, our results preclude the usefulness of preoperative AAM profile for predicting long term weight status after sleeve gastrectomy. Sixthly, our results should be interpreted with caution for a potential type I error induced by multiple comparisons.

## 5. Conclusions

In conclusion, pre-operative profiles of AAMs (especially those of serotonin and serotonin/5-HTrp ratio) showed superior predictive performances for weight status three and six months after sleeve gastrectomy. Further studies are warranted to assess whether measurements of serum AAMs might assist in the identification of patients who maintain successful weight loss in long-term follow-ups and to elucidate the biological mechanisms by which certain AAMs might mediate successful weight loss after sleeve gastrectomy.

## Figures and Tables

**Table 1:** Baseline characteristics.

Variables	Values (*n* = 27)
Age, years	42.1 ± 12.9
Female sex, *n* (%)	17 (63)
Body mass index^1^, kg/m^2^	38.7 ± 5.2
Body weight, kg	105.1 ± 17.0
Waist circumference, cm	120.5 ± 19.9
Current smoker, *n* (%)	4 (15)
Metabolic syndrome, *n* (%)	21 (78)
Hypertension, *n* (%)	14 (52)
Diabetes, *n* (%)	20 (74)
Dyslipidemia, *n* (%)	20 (74)

Values are given as mean ± standard deviation ^1^ The body mass index is the weight in kilograms divided by the square of the height in meters.

**Table 2:** Average values and percentage changes at three and six months after sleeve gastrectomy.

Variables	Measurement Time						
	Baseline (*n* = 27)	3 Months after Surgery (*n* = 27)			6 Months after Surgery (*n* = 27)		
	Values	Values	Change from Baseline, %	*p*	Values	Change from Baseline, %	*p*
Body-mass index, kg/m^2^	38.7 ± 5.2	31.5 ± 5.1	−18.8 ± 4.2	<0.001	27.9 ± 4.6	−26.1 ± 4.6	<0.001
Body weight, kg	105.1 ± 17.0	85.1 ± 15.3	−18.8 ± 4.2	<0.001	73.9 ± 11.5	−26.1 ± 4.6	<0.001
% Excess weight loss		62.5 ± 35.1			92.3 ± 49.8		
Waist circumference, cm	120.5 ± 19.9	102.3 ± 13.6	−14.9 ± 7.4	<0.001 *	94.4 ± 13.5	−21.7 ± 7.3	<0.001 *
Body fat mass, kg	54.4 ± 12.0	35.5 ± 10.0	−28.7 ± 8.0	<0.001	26.0 ± 6.7	−46.5 ± 8.1	0.002
Fat free mass, kg	57.5 ± 9.9	50.3 ± 8.3	−10.3 ± 4.2	<0.001	48.6 ± 9.8	−12.0 ± 6.5	0.007
Fasting plasma glucose, mg/dL	131.6 ± 54.4	104.2 ± 17.6	−14.5 ± 33.3	0.028*	100.6 ± 18.6	−17.0 ± 29.2	0.034 *
HOMA-IR	6.4 ± 10.0	2.8 ± 1.6	−50.6 ± 36.4	0.033	2.2 ± 0.6	−47.1 ± 50.8	0.044
Systolic blood pressure, mmHg	142.6 ± 5.8	134.3 ± 6.3	5.7 ± 2.2	<0.001	126.1 ± 4.8	11.6 ± 2.2	<0.001
Diastolic blood pressure, mmHg	97.6 ± 6.2	89.3 ± 5.4	8.4 ± 3.2	<0.001	83.1 ± 5.7	14.9 ± 3.2	<0.001
High-density lipoprotein cholesterol, mg/dL	46.0 ± 10.4	49.2 ± 10.0	9.9 ± 31.6	0.792 *	54.6 ± 14.4	15.6 ± 20.9	0.041 *
Triglycerides, mg/dL	173.6 ± 82.2	131.3 ± 37.1	−10.7 ± 42.3	0.042 *	115.6 ± 37.0	−23.6 ± 31.7	0.016 *

HOMA-IR, Homeostasis model assessment-insulin resistance. Values are given as mean ± standard deviation. Body fat mass and fat free mass were measured with bioimpedance analyses. * *p*-Value was calculated with Wilcoxon signed rank test.

**Table 3:** Association between baseline amino acid metabolites and %EWL at three and six months after sleeve gastrectomy.

	%EWL at 3 Months (*n* = 27)		%EWL at 6 Months (*n* = 27)	
	β (SE)	*p*	β (SE)	*p*
**Branched-chain amino acids**				
Leucine	0.24 (0.32)	0.455	0.26 (0.47)	0.576
Isoleucine	0.67 (0.27)	0.026	1.02 (0.36)	0.016
Valine	0.17 (0.19)	0.378	0.31 (0.28)	0.281
**Aromatic amino acids**				
Tryptophan	−0.57 (0.40)	0.169	−1.21 (0.59)	0.061
Phenylalanine	0.04 (0.48)	0.921	0.02 (0.76)	0.973
Tyrosine	−0.37 (0.26)	0.171	−0.52 (0.39)	0.208
**Sum of branched-chain amino acids**	0.13 (0.09)	0.181	0.20 (0.13)	0.155
**Sum of large neutral amino acids**	−0.15 (0.13)	0.273	0.04 (0.10)	0.673
**Metabolites from serotonin pathway**				
5-hydroxytryptophan *	61.0 (37.8)	0.124	71.7 (62.6)	0.273
Serotonin	−39.7 (13.5)	0.009	−50.8 (21.1)	0.032
5-hydroxyindoleacetic acid *	106.7 (32.5)	0.004	125.2 (54.7)	0.040
**Ratios of metabolites from serotonin pathway**				
5-hydroxytryptophan / Tryptophan ^†^	64.6 (30.6)	0.049	83.2 (45.8)	0.093
Serotonin / 5-hydroxytryptophan ^‡^	−45.7 (14.1)	0.005	−45.2 (23.1)	0.073
5- hydroxy-indoleacetic acid / Serotonin ^§^	54.7 (12.4)	<0.001	54.5 (21.6)	0.026

%EWL, % excess weight loss; SE, standard error. Linear regression models were adjusted for baseline body mass index. %EWL was calculated by dividing the number of kilograms lost by the number of kilograms in a patient’s excess body weight. Large neutral amino acids are a sum of branched-chain amino acids and aromatic amino acids. Results of metabolites from kynurenine, indole, and tyrosine pathway are shown in Supplemental Table S4. All simbols mean the same and significant association with %EWL

## Supplementary Materials

The following are available online at https://www.mdpi.com/2077-0383/9/8/2348/s1, Figure S1: Amino acid metabolites analyzed in the current study, Table S1: Inclusion and exclusion criteria, Table S2: Protocol for measurement of serum amino acids metabolites, Table S3: Chromatographic Retention time (RT), selected MRM parameters, DP, EP, CE, CXP for each analyte measured, Table S4: Association between baseline amino acid metabolites and %EWL at 3 and 6 month after sleeve gastrectomy, Table S5: Sensitivity analyses: association between baseline serotonin and serotonin/5-hydroxytryptophan ratio, and %EWL after sleeve gastrectomy.
