# Supplementary material for: Amino Acid Metabolites and Slow Weight Loss in the Early Postoperative Period after Sleeve Gastrectomy
Source: J Clin Med. 2020 Jul 23;9(8):2348. doi: 10.3390/jcm9082348 (PMC7463855; doi:10.3390/jcm9082348)
Supplement: Supplementary file 1 [file jcm-09-02348-s001.pdf]

Supplementary Materials

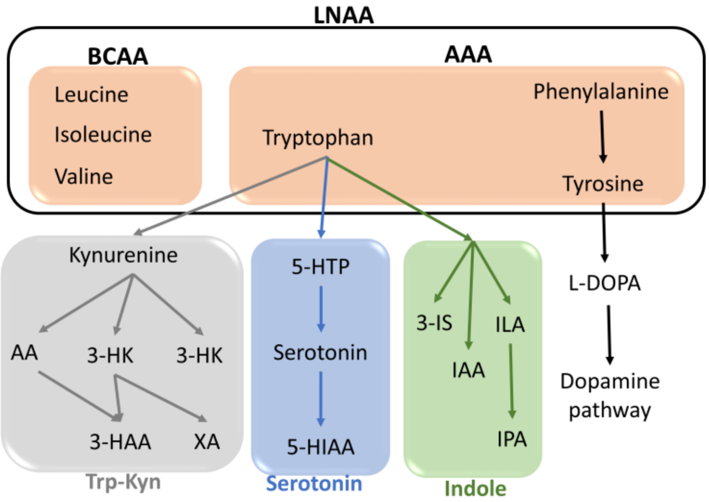

Figure S1. Amino acid metabolites analyzed in the current study.

Table S1. Inclusion and exclusion criteria

Inclusion criteria	Entry into the study would require that the patient:<br>1. ≥ 20 years old<br>2. body mass index ≥ 35 kg/m^2, or a body mass index ≥ 30 kg/m^2 and at least one or more obesity-related co-morbidities.
Exclusion criteria	1. Prior complex gastrointestinal surgery, including that of the stomach, small intestine, large intestine, bile duct, pancreas, and spleen; Nissen and trauma (except hemorrhoidectomy, herniorrhaphy, and appendectomy);<br>2. abdominal, thoracic, pelvic, and/or obstetric-gynecologic surgery within 3 months or at the discretion of the Investigator;<br>3. cardiovascular conditions including significant known coronary artery disease (CAD), uncompensated congestive heart failure, history of stroke, or uncontrolled hypertension (defined as medially treated with the mean of three separate measurements SBP >180 mmHg or DBP >110 mmHg). Subjects with CAD who were successfully treated with coronary artery bypass grafts or percutaneous coronary interventions within 3 months and who have no evidence of active ischemia are eligible;<br>4. kidney diseases including renovascular hypertension, renal artery stenosis, or end-stage renal disease;<br>5. known history of chronic liver diseases including liver cirrhosis and alpha-1-antitrypsin deficiency;<br>6. gastrointestinal disorders including a known history of celiac disease or any other malabsorptive disorders or inflammatory bowel disease (Crohn's disease or ulcerative colitis);<br>7. psychiatric disorders including dementia, active psychosis, history of suicide attempts, and alcohol or drug abuse within 12 months;<br>8. severe pulmonary disease defined as forced expiratory volume-one second (FEV-1) < 50% of the predicted value;<br>9. anemia defined as hemoglobin < 8 g/dL in females and 10 g/dL in males;<br>10. malignancy within 5 years (except squamous cell and basal cell cancer of the skin). Patients diagnosed with early or stage 1 cancer that was successfully treated are eligible based on the Investigator's discretion;<br>11. any condition or major illness that in the Investigator's judgement places the subject at undue risk by participating in the study;<br>12. pregnancy;<br>13. unable to understand the risks, realistic benefits, and compliance requirements of each program;<br>14. use of investigational therapy or participation in any other clinical trial within 3 months; and<br>15. plans to move outside South Korea within the next 2 years.

**Table S2.** Protocol for measurement of serum amino acids metabolites
1. Amino acids and their metabolites were quantified using liquid chromatography-mass spectrometry (LC-MS) based on previous publication methods[1,2]. Briefly, 10 μL of ^{13}C-tryptophan (5 μM), as the internal standard (IS), was added to 100 μL of serum and standard solution in 4% bovine serum albumin solution and diluted with 20 μL of water containing 0.1% formic acid (v/v). Subsequently, 400 μL of cold acetonitrile for protein precipitation was added, vortexed, centrifuged at 10,000 x g at 4°C for 10 min. Next, 400 μl of the supernatant was dried using a speed vacuum evaporator and reconstituted in 0.1% formic acid in water/acetonitrile (9/1, v/v) solution. Then, 10 μL of the samples were injected into an HPLC-MS/MS system (Agilent 1200 HPLC equipped with AB SCIex 3200 Mass analyzer) equipped with the Waters Atlantis T3 (4.6×150 mm i.d., 3 μm) column at 30°C.
2. Ions of each analyzed compound, except the IS, were detected in a positive ionization mode using the multiple reaction monitoring mode. Liquid chromatographic separation was performed with mobile phases A (0.1% formic acid in water, v/v) and B (0.1% formic acid in acetonitrile, v/v) at 0.5 mL/min flow rate under the following conditions: 5% for the first gradient starting at 5% B to 40% B in 6 min, to 90% B in 5 min, staying at 90% B for 5 min, then to 5% B in 1 min. The column equilibration was performed for 8 min before each analysis under the 5% B condition.
3. In terms of the MS conditions, the ion source temperature was set to 600°C. The curtain and nebulizer gas were set at 20 psi and 50 psi, respectively. The MS capillary voltage was 4.5 kV for the positive mode or -4.5 kV for the negative mode. The acquired data were analyzed using Analyst software (v1.6.3, SCIex, USA).
4. In addition to metabolite measurements, we calculated ratios between adjacent metabolites to compare the activity of enzymes among participants: 5-HTP/ tryptophan ratio for tryptophan hydroxylase (TPH), serotonin/5-HTP ratio for aromatic L-amino acid decarboxylase (AADC), 5-HIAA/serotonin ratio for monoamine oxidase A (MAO-A), kynurenine/tryptophan ratio for tryptophan 2,3-dioxygenase (TDO), AA/kynurenine ratio for kynureninase (KYNU), 3-HK/kynurenine ratio for kynurenine 3-monooxygenase (KMO), 3-HAA/3-HK ratio for kynureninase (KYNU), KA/kynurenine ratio for kynurenine aminotransferase (KAT), XA/3-HK ratio for kynurenine aminotransferase (KAT), tyrosine/phenylalanine ratio for p-hydroxyphenylacetaldehyde (PHA), and LDOPA/tyrosine ratio for tyrosine hydroxylase (TH).

[1] Zhu W, Stevens AP, Dettmer K, et al. Quantitative profiling of tryptophan metabolites in serum, urine, and cell culture supernatants by liquid chromatography-tandem mass spectrometry. Anal Bioanal Chem 2011;401:3249-3261.

[2] Choi JM, Park WS, Song KY, et al. Development of simultaneous analysis of tryptophan metabolites in serum and gastric juice - an investigation towards establishing a biomarker test for gastric cancer diagnosis. Biomed Chromatogr 2016;30:1963-1974.

Table S3. Chromatographic Retention time (RT), selected MRM parameters, DP, EP, CE, CXP for each analyte measured

Compounds	Exact Mass	RT	Ion mode	[MH+] or [M-H]-	Product Ion Mass	DP	EP	CE	CXP
Leucine ($C_6H_{13}NO_2$)	131.095	6.5	Pos	132.1	**43.2**	21	7.5	31.	10
					86.1	21	7.5	31.0	10
Isoleucine ($C_6H_{13}NO_2$)	131.095	6.2	Pos	132.0	**86.1**	26	5.5	21.0	10
Valine ($C_5H_{11}NO_2$)	117.0789	4.1	Pos	118.1	**72.1**	26	5.0	19.0	10
Phenylalanine ($C_9H_{11}NO_2$)	165.079	7.3	Pos	166.2	103.2	21	3.0	33.0	10
					**120.2**	21	3.0	19.0	10
Tyrosine ($C_9H_{11}NO_3$)	181.074	6.3	Pos	181.9	**91.2**	26	6.0	35.0	14
					136.1	26	5.5	35.0	14
Tryptophan ($C_{11}H_{12}N_2O_2$)	204.09	8.1	Pos	205.0	**117.9**	26	6.0	33.0	12
					158.8	26	6.0	33.0	12
Kynurenine ($C_{10}H_{12}N_2O_3$)	208.0848	7.4	Pos	209.1	94.0	26	5.0	19.0	10
					**192.200**	26	5.0	13.0	10.0
Kynurenic acid ($C_{10}H_7NO_3$)	189.043	9.2	Pos	189.900	**116.000**	31	3.0	39.0	12.0
					143.900	21	3.0	39.0	12.0
Anthranilic acid ($C_7H_7NO_2$)	137.048	11.4	Pos	138.106	92.100	16	3.0	27.0	14.0
					**119.900**	16	3.0	15.0	14.0
Xanthurenic acid ($C_{10}H_7NO_4$)	205.0375	8.1	Pos	206.071	**132.200**	51	10.5	41.0	12.0
					159.900	51	10.5	23.0	12.0
3-Hyroxykynurenine ($C_{10}H_{12}N_2O_4$)	224.0797	6.2	Pos	225.061	110.100	26	8.5	23.0	20.0
					**208.100**	26	8.5	13.0	20.0
3-Hydroxyanthranilic acid ($C_7H_7NO_3$)	153.043	9.0	Pos	154.000	108.100	26	2.5	25.0	10.0
					**136.200**	21	2.0	25.0	10.0
5-hydroxyindoleacetic acid ($C_{10}H_9NO_3$)	191.058	9.6	Pos	192.100	**146.100**	26.0	5.5	29.0	10.0
5-Hydroxytryptophan ($C_{11}H_{12}N_2O_3$)	220.084	6.9	Pos	221.100	**175.000**	26.0	5.5	23.0	12.0
L-Dihydroxyphenylalanine ($C_9H_{11}NO_4$)	197.069	5.0	Pos	197.934	107.200	36.0	4.0	29.0	14.0
					**152.000**	36.0	4.0	21.0	14.0
Indole-3-acetic acid ($C_{10}H_9NO_2$)	175.063	11.8	Pos	176.100	**130.100**	26.0	4.5	21.0	10.0
Indole-3-propionic acid ($C_{11}H_{11}NO_2$)	189.079	12.4	Pos	190.100	**129.800**	16.0	3.5	17.0	10.0
					172.100	16.0	3.0	17.0	10.0
Indole-3-lactic acid ($C_{11}H_{11}NO_3$)	205.074	11.1	Pos	206.100	**130.000**	21.0	3.5	25.0	12.0
					187.800	26.0	3.5	25.0	12.0
Indoxyl sulfate ($C_8H_7NO_4S$)	213.01	10.8	Neg	211.919	**79.800**	-65.0	-1.0	-40.0	-16.0
					131.800	-65.0	-1.0	-28.0	-16.0
IS(^{13}C-Tryptophan) ($C_{11}H_{12}N_2O_2$)	215.09	8.3	Pos	216.100	126.100	26.0	5.5	33.0	12.0
			Neg	214.000	124.100	-50.0	-10.0	-24.0	-12.0

MRM: multiple reaction mode, DP: declustering potential, EP: entrance potential, CE: collision energy, CXP: collision cell exit potential, IS: internal standard, [MH+]: positive ion mode, [M-H]-: negative ion mode

Table S4. Association between baseline amino acid metabolites and %EWL at 3 and 6 month after sleeve gastrectomy

	**%EWL at 3 months**		**%EWL at 6 months**	
	**β (SE)**	**P-value**	**β (SE)**	**P-value**
**Metabolites from kynurenine pathway**				
Kynurenine*	42.3 (45.6)	0.366	83.4 (77.0)	0.299
Anthranilic acid	-159.3 (308.6)	0.612	-515.7 (481.4)	0.304
3-Hydroxykynurenine	323.0 (302.5)	0.300	520.3 (511.5)	0.328
3-Hydroxyanthranilic acid*	-32.6 (49.2)	0.516	-84.7 (74.7)	0.278
Kynurenic acid*	26.3 (25.6)	0.318	59.8 (40.5)	0.164
Xanthurenic acid*	-7.4 (29.2)	0.803	-17.8 (44.8)	0.697
**Metabolites from indole pathway**				
Indoxyl sulfate	1.11 (2.25)	0.628	1.10 (4.40)	0.806
Indole-3-acetic acid	0.57 (4.86)	0.907	-4.54 (8.69)	0.610
Indole-3-lactic acid*	38.54 (31.61)	0.239	58.22 (52.34)	0.286
Indole-3-propionic acid*	-8.47 (6.20)	0.189	-15.81 (13.14)	0.251
**Metabolites from tyrosine pathway**				
L-Dihydroxyphenylalanine	-106 (764)	0.891	-721 (1119)	0.531

%EWL: % excess weight loss, SE: standard error

Regression models were adjusted for baseline body mass index. %EWL was calculated by dividing the number of kilograms lost by the number of kilograms in a patient's excess body weight.

*Data were log-transformed to increase normality before analyses

Table S5. Sensitivity analyses: association between baseline serotonin and serotonin/5-hydroxytryptophan ratio, and %EWL after sleeve gastrectomy

	n	**Serotonin**			
		**3 months**		**6 months**	
		**β (SE)**	**P-value**	**β (SE)**	**P-value**
Age, ≥ 45 yrs	11	-71.2 (28.7)	0.038	-51.2 (21.7)	0.036
Female	14	-38.4 (14.1)	0.020	-38.5 (17.5)	0.056
Body mass index, ≥ 35 kg/m^2	14	-23.4 (9.1)	0.027	-30.6 (13.4)	0.041
Hypertension	13	-72.8 (27.2)	0.023	-60.9 (54.1)	0.013
Diabetes	15	-39.1 (16.0)	0.031	-42.0 (28.1)	0.070
Non-smoker	18	-27.3 (9.6)	0.013	-31.5 (14.8)	0.060
		**Serotonin / 5-hydroxytryptophan ratio**			
	**n**	**3 months**		**6 months**	
		**β (SE)**	**P-value**	**β (SE)**	**P-value**
Age ≥ 45 yrs	11	-85.9 (37.0)	0.049	-54.3 (26.6)	0.064
Female	14	-50.2 (18.4)	0.020	-46.8 (22.1)	0.063
Body mass index, ≥ 35 kg/m^2	14	-22.2 (10.0)	0.049	-54.4 (25.7)	0.054
Hypertension	13	-75.7 (38.5)	0.078	-52.9 (73.4)	0.191
Diabetes	15	-40.1 (19.3)	0.060	-44.0 (33.2)	0.117
Non-smoker	18	-30.1 (12.1)	0.026	-31.2 (18.3)	0.120

%EWL, % excess weight loss; *SE*, standard error. Regression models were adjusted for baseline body mass index. %EWL was calculated by dividing the number of kilograms lost by the number of kilograms in a patient's excess body weight.
